# Predicting rTMS Treatment Response in Major Depression Using Minimal-Electrode EEG and Machine Learning

**DOI:** 10.1192/j.eurpsy.2026.11755

**Published:** 2026-07-31

**Authors:** C.-H. Chang, A. T. Sack, C. M. Chu, H.-A. Chang

**Affiliations:** 1 Psychiatry, An Nan Hospital, China Medical University, Tainan, Taiwan; 2 Cognitive Neuroscience, Maastricht University, Maastricht, Netherlands; 3 Psychiatry, Kaohsiung Veterans General Hospital, Kaohsiung; 4 Psychiatry, Tri-Service General Hospital, National Defense Medical University, Taipei, Taiwan

## Abstract

**Introduction:**

Repetitive transcranial magnetic stimulation (rTMS) is an established therapy for treatment-resistant depression, yet response variability limits its clinical efficiency. Resting-state electroencephalography (EEG), offering accessible neural biomarkers, may enhance individualized prediction of treatment outcomes through machine learning integration.

**Objectives:**

This study aimed to determine whether a reduced 8-electrode EEG configuration could predict rTMS treatment response as accurately as a 30-electrode system, using nonlinear EEG features and optimized classification algorithms.

**Methods:**

A total of 100 patients with major depressive disorder underwent pre-treatment EEG before a two-week accelerated intermittent theta-burst rTMS protocol (1800 pulses/session, 3 sessions/day separated by 60 mins for 10 working days). EEG features—including band power, coherence, phase-lag index (PLI), and phase-locking value (PLV)—were extracted. A two-stage feature selection process (recursive feature elimination and sequential backward selection) preceded linear discriminant analysis (LDA). Predictive accuracy was assessed by 5-fold cross-validation and 1,000 shuffle iterations.

**Results:**

Multi-feature EEG models consistently outperformed single-feature analyses in predicting rTMS treatment outcomes. The 8-electrode model achieved 78.9% accuracy and an AUC of 73.3%, comparable to or surpassing the 30-electrode configuration. PLI was the most robust single predictor, while combining multiple features markedly enhanced classification performance. Among six tested classifiers, LDA yielded the most stable and generalizable results, particularly with limited training data, underscoring its clinical applicability.

**Conclusions:**

Resting-state EEG combined with machine learning can reliably predict rTMS outcomes in depression. Importantly, high accuracy was achieved using a minimal 8-electrode montage, highlighting the clinical feasibility of low-cost, rapid EEG applications for personalized neuromodulation strategies.

**Disclosure of Interest:**

C.-H. Chang Grant / Research support from: An Nan Hospital, China Medical University Hospital (ANHRF113-07 and ANHRF113-53), A. Sack Grant / Research support from: ATS received funding from the Dutch Research Council (NWO)., Consultant of: ATS is director of the Academy of Brain Stimulation (www.brainstimulation-academy.com) and the International Clinical TMS Certification Course (www.tmscourse.eu), receiving equipment support from MagVenture, Magstim, DEYMED, Yingchi, BrainsWay., C. Chu: None Declared, H.-A. Chang Grant / Research support from: Advanced National Defense Technology & Research Program, National Science and Technology Council of Taiwanese Government (NSTC-112-2314-B-016-017-MY3), Tri-Service General Hospital (TSGH_D_114143 and TSGH-B-114024), Medical Affairs Bureau, Ministry of National Defense, Taipei, Taiwan (MND-MAB-D-114127 and MND-MAB-D-114125),

